# Emerging and Neglected Viruses of Zoonotic Importance in Croatia

**DOI:** 10.3390/pathogens10010073

**Published:** 2021-01-15

**Authors:** Tatjana Vilibic-Cavlek, Ljubo Barbic, Anna Mrzljak, Dragan Brnic, Ana Klobucar, Maja Ilic, Natasa Janev-Holcer, Maja Bogdanic, Lorena Jemersic, Vladimir Stevanovic, Irena Tabain, Stjepan Krcmar, Marko Vucelja, Jelena Prpic, Marko Boljfetic, Pavle Jelicic, Josip Madic, Ivana Ferencak, Vladimir Savic

**Affiliations:** 1 Department of Virology, Croatian Institute of Public Health, 10000 Zagreb, Croatia; maja.bogdanic11@gmail.com (M.B.); irena.tabain@hzjz.hr (I.T.); ivana.ferencak@hzjz.hr (I.F.); 2 School of Medicine, University of Zagreb, 10000 Zagreb, Croatia; anna.mrzljak@gmail.com; 3 Department of Microbiology and Infectious Diseases with Clinic, Faculty of Veterinary Medicine, University of Zagreb, 10000 Zagreb, Croatia; ljubo.barbic@vef.hr (L.B.); vladimir.stevanovic@vef.hr (V.S.); josip.madic@vef.hr (J.M.); 4 Department of Medicine, Merkur University Hospital, 10000 Zagreb, Croatia; 5 Department of Virology, Croatian Veterinary Institute, 10000 Zagreb, Croatia; brnic@veinst.hr (D.B.); jemersic@veinst.hr (L.J.); balatinec@veinst.hr (J.P.); 6 Department of Epidemiology, Andrija Stampar Institute of Public Health, 10000 Zagreb, Croatia; ana.klobucar@stampar.hr; 7 Department of Epidemiology, Croatian Institute of Public Health, 10000 Zagreb, Croatia; maja.ilic@hzjz.hr; 8 Environmental Health Department, Croatian Institute of Public Health, 10000 Zagreb, Croatia; natasa.janev@hzjz.hr (N.J.-H.); pavle.jelicic@hzjz.hr (P.J.); 9 Department of Biology, Josip Juraj Strossmayer University of Osijek, 31000 Osijek, Croatia; stjepan@biologija.unios.hr; 10 Faculty of Forestry, University of Zagreb, 10000 Zagreb, Croatia; marko.vucelja@sumfak.unizg.hr (M.V.); marko.boljfetic@sumfak.unizg.hr (M.B.); 11 Poultry Center, Croatian Veterinary Institute, 10000 Zagreb, Croatia; savic@veinst.hr

**Keywords:** arboviruses, rodent-borne viruses, hepatitis E virus, SARS-CoV-2, rotaviruses, rabies virus, epidemiology, Croatia

## Abstract

Several arboviruses have emerged in Croatia in recent years. Tick-borne encephalitis is endemic in continental counties; however, new natural micro-foci have been detected. Two autochthonous dengue cases were reported in 2010. West Nile virus emerged in 2012, followed by emergence of Usutu virus in 2013. Although high seroprevalence rates of Toscana virus have been detected among residents of Croatian littoral, the virus remains neglected, with only a few clinical cases of neuroinvasive infections reported. Lymphocytic choriomeningitis virus is a neglected neuroinvasive rodent-borne virus. So far, there are no reports on human clinical cases; however, the seroprevalence studies indicate the virus presence in the Croatian mainland. Puumala and Dobrava hantaviruses are widely distributing rodent-borne viruses with sporadic and epidemic occurrence. Hepatitis E virus is an emerging food-borne virus in Croatia. After the emergence in 2012, cases were regularly recorded. Seropositivity varies greatly by region and population group. Rotaviruses represent a significant healthcare burden since rotavirus vaccination is not included in the Croatian national immunization program. Additionally, rotaviruses are widely distributed in the Croatian ecosystem. A novel coronavirus, SARS-CoV-2, emerged in February 2020 and spread rapidly throughout the country. This review focuses on emerging and neglected viruses of zoonotic importance detected in Croatia.

## 1. Zoonotic Viruses in Croatia

Arboviruses represent an emerging public health problem worldwide. Tick-borne encephalitis virus (TBEV) and West Nile virus (WNV) are nowadays the most widely distributed arboviruses. Although many human TBEV and WNV infections are asymptomatic or present as a non-specific febrile disease, the severe neuroinvasive disease may occur. Other medically important arboviruses such as dengue (DENV), chikungunya (CHIKV), and Zika virus (ZIKV) are generally associated with mild clinical symptoms; however, the more severe form (dengue hemorrhagic fever/shock syndrome) has also been observed, especially during epidemics.

Hantaviruses are globally distributed rodent-borne viruses. Many other zoonotic viruses are largely neglected despite their potential to emerge globally [1].

In the past decade, several arboviruses have (re-)emerged in Croatia. New natural micro-foci of TBEV have been detected in continental Croatian counties [2,3,4]. In 2010, two autochthonous cases of DENV infection were reported [5], followed by detection of first human cases of WNV neuroinvasive disease in 2012 [6]. Usutu virus is another neuroinvasive flavivirus detected in Croatia in 2013 [7,8]. Sporadic imported cases CHIKV and ZIKV infection have been continuously notified in returning travelers [9,10]. Although seroprevalence of Toscana virus (TOSV) is high among residents of the Croatian littoral [11], the virus remains neglected since clinical cases of neuroinvasive infection are diagnosed only sporadically [12,13]. Other arboviruses such as sandfly fever Sicilian (SFSV) and Naples virus (SFNV), Tahyna (TAHV), Bhanja virus (BHAV), and Čalovo virus have also been confirmed serologically, especially among inhabitants of the Croatian islands. Three laboratory BHAV infections as well as one natural infection detected in a patient with meningoencephalitis and spastic quadriparesis have also been reported [14,15,16]. Lymphocytic choriomeningitis virus (LCMV) is a neglected neuroinvasive rodent-borne virus. The seroprevalence studies indicate that LCMV is present in continental Croatian regions [17]. Hantaviruses Puumala (PUUV) and Dobrava (DOBV) are rodent-borne viruses causing hemorrhagic fever with renal syndrome (HFRS). Both viruses are detected in humans in Croatia [18,19,20,21,22], while Saarema (SAAV) and Tula virus (TULV) have also been detected in rodents [23,24]. Hepatitis E virus (HEV) is an emerging food-borne zoonotic virus in Croatia. Seroprevalence rates vary greatly by region and population group [25,26,27]. Rotaviruses, most notably the species rotavirus A (RVA), represent a significant healthcare burden since RVA vaccination is not included in the Croatian national immunization program [28]. RVA is widely distributed in the Croatian ecosystem; however, the importance of zoonotic transmission of RVAs might be underestimated due to the focus on RVA strain surveillance in clinically ill patients combined with lower virulence of animal RVAs in humans [29,30,31]. A novel coronavirus, severe acute respiratory syndrome coronavirus 2 (SARS-CoV-2), is a respiratory virus that emerged in February 2020 and spread rapidly throughout the country. Acute infections as well as seropositivity have been confirmed in humans and pet animals [32,33,34]. The rabies virus (RABV) still presents a major veterinary and public health threat. The last case of human rabies in Croatia was reported in the 1960s [35]; however, epidemiological data have confirmed the presence of rabies virus (RABV) in wildlife since 1977 [36]. After starting the oral rabies vaccination (ORV) campaign in 2011, the last wildlife rabies positive case was confirmed in February 2014 [37].

This review focuses on the epidemiology, clinical aspects, and molecular epidemiology of emerging and neglected viruses of zoonotic importance detected in Croatia (Table 1).

## 2. Arthropod-Borne Viruses

**Figure 1:**
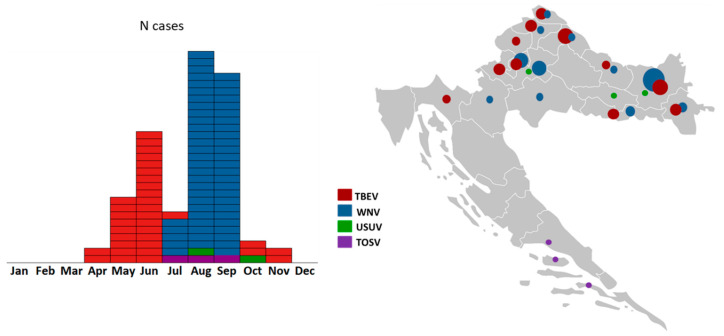
Seasonal (left) and geographic distribution (right) of neuroinvasive arboviral infections in Croatia (2017–2020). Within the arbovirus surveillance program (project CRONEUROARBO), four neuroinvasive arboviruses were detected: TBEV, WNV, USUV, and TOSV. Circle size corresponds to the number of reported human cases.

After detection of the first WNV cases in 2012, a vector-borne flaviviruses national surveillance program has been established in Croatia. Additionally, the invasive mosquito species monitoring has been implemented and continuously conducted since 2016. The aim was to collect data and create a distribution map of the invasive mosquito species and a unique national database to conduct a risk assessment for vector-borne diseases. Moreover, from 2017 to 2020, a project on the prevalence and molecular epidemiology of emerging and re-emerging arboviruses (CRONEUROARBO) was conducted. This interdisciplinary study (‘One Health’) included patients with neuroinvasive infection as well as the screening of sentinel animals (horses, wild birds, poultry) and vectors (mosquitoes and ticks). A total of 474 patients with the neuroinvasive disease were included in the study. Arboviral etiology was confirmed in 114 (24.1%) patients: WNV in 62 (13.1%), TBEV in 46 (9.7%), USUV in 3 (0.6%), and TOSV in 3 (0.6%) patients [3,4,13,44,74,75,76,77]. Seasonal and geographic distribution of arbovirus infections are presented in Figure 1.

### 2.1. Tick-Borne Encephalitis Virus

**Figure 2:**
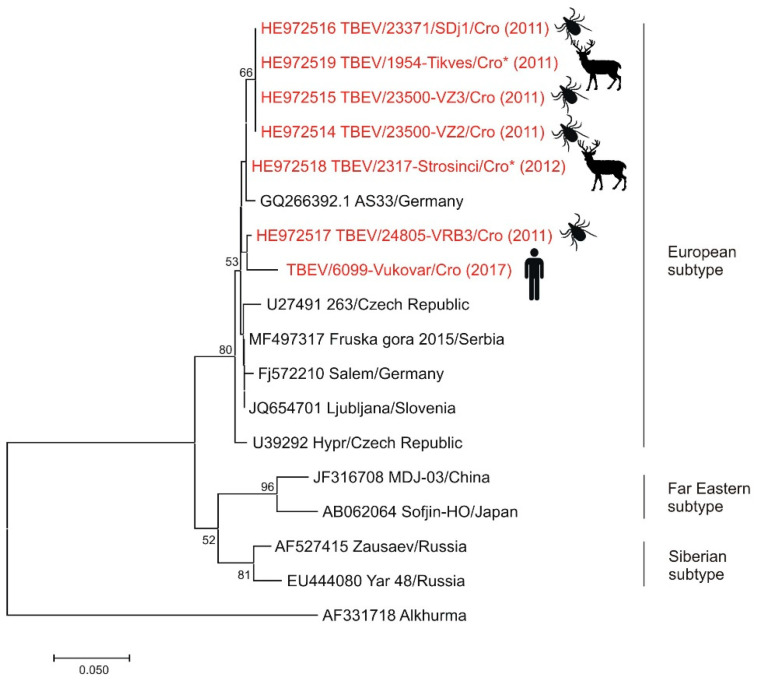
Phylogenetic neighbor-joining analysis of a 174-nucleotide fragment of the TBEV 5′-NTR and partial capsid gene. Strains detected in Croatia are marked in red with year of detection in parentheses and indicated figure of the host. Alkhurma virus was included as an outgroup. GenBank accession number for each isolate used in the analysis is specified. Supporting (≥50%) bootstrap values of 1000 replicates are displayed at the nodes. Horizontal distances are proportional to genetic distance. Scale bar indicates nucleotide substitutions per site.

TBE is endemic in continental Croatia, expanding its area of circulation in recent years with the emergence in several new natural foci [4,78]. Small foci of TBEV have also been identified in Middle and South Adriatic [14,60,79]. Endemicity is highest in northwestern counties with a mean incidence of 3.61–6.78/100,000 inhabitants [1]. The number of reported human TBE cases (2007–2019) ranged from six cases in 2016 to 45 in 2012 [80]. In 2007–2008, samples from small wild rodents (*A. flavicollis*, *A. agrarius*, *Myodes glareolus*) captured at two different sites (mountainous and lowland region) in Croatia were tested for the presence of TBEV antibodies and RNA; however, no one tested positive [81]. In a study published in 2011, TBEV IgG antibodies were detected in 4.41% forestry workers from the Croatian central Posavina (Sava River Basin). Although no significant differences were found in the TBEV seroprevalence according to different risk factors, certain factors with a higher odds ratio such as cutting trees in the forest, afforestation, contact with wild animals, and hunting should be taken into account as possible risk factors for TBE [38]. In 2015, an outbreak of TBE associated with raw goat milk consumption was reported [39]. In 2019, a cluster of patients with TBE was detected in a new micro-focus in the Gorski Kotar region. Five of the six patients reported consuming raw (unpasteurized) goat milk from the same farm in the two-week period before symptom onset. Milk samples from 12 goats from the implicated farm were tested for the TBEV using RT-PCR. Although TBEV RNA was not detected in the milk, serological testing of goats and other farm animals (horses, dog) yielded evidence of exposure to the virus. Six goats from the flock showed high titers of TBEV neutralizing antibodies suggesting that the vehicle for the outbreak was raw goat milk from the farm [4]. A seroprevalence study conducted in 2016 detected TBEV IgG antibodies in 3.65% of dogs from eastern Croatia [40]. In 2017, TBEV IgG antibodies were identified in 14.3% of sentinel horses from the endemic regions of the Croatian mainland [41]. A study on the genetic characterization of TBEV was conducted in 2011. Ticks (*n* = 371) removed from hunted red foxes (*Vulpes vulpes*) and spleen samples from hunted red deer (*Cervus elaphus*) were tested for the presence of TBEV RNA. TBEV RNA was detected in 1.6% *Ixodes ricinus* (Linnaeus, 1758) and *Ixodes hexagonus* (Leach, 1815) ticks and two (1.1%) spleen samples from red deer. Additionally, in 2017, TBEV RNA was detected in one urine sample from a patient with severe TBEV neuroinvasive infection. Croatian TBEV isolates were shown to be closely related, all belonging to the European subtype TBEV (Figure 2) [42,43]. However, based on nucleotide and amino acid sequence analysis, two clusters were identified [42]. The presented data showed that TBE is widespread in the continental Croatian regions.

### 2.2. Dengue Virus

Although a seroepidemiological study conducted in 1980 in a limited area of northeastern Croatia proved the presence of antibodies to DENV type 1 and 2 in 2.1% and 3.9% healthy young inhabitants [51], no cases of dengue were registered by the health services until 2010. After information about a dengue case in a German tourist acquired in Croatia, health professionals were alerted to assess the situation, resulting in the diagnosis of the second case of autochthonous dengue fever in a resident of the Peljesac peninsula (South Dalmatia), the same area where the German patient had stayed as well as detection of 15 persons with serologic evidence of recent dengue infection [5]. The sequences from two patients were identified as DENV type 1 [52]. During 2011–2012, a seroprevalence and entomological study was conducted. A total of 1180 serum samples were collected from residents of seven counties at the Croatian littoral and four counties in northeastern Croatia. Seven samples (0.59%) tested positive for DENV antibodies. Seroprevalence rates varied from 0 to 2.21% with the highest seropositivity (2.21%) in the Dubrovnik-Neretva County, where autochthonous dengue cases were recorded in 2010. In addition, 3699 mosquitoes were collected from 126 localities along the Adriatic coast in August–September, 2011. *Aedes albopictus* was the most prevalent species (81.37%). DENV RNA was not detected by RT-PCR among 1748 female mosquitoes [53]. Sporadic imported dengue cases were continuously notified in travelers returning from endemic areas [82].

### 2.3. West Nile Virus

**Figure 3:**
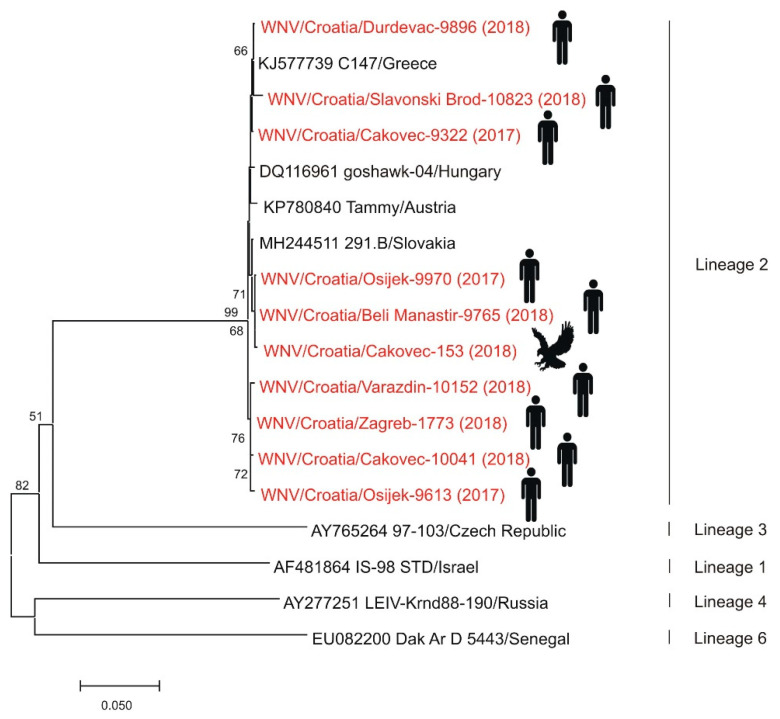
Phylogenetic neighbor-joining analysis of a 848-nucleotide fragment of the WNV NS5 gene. Strains detected in Croatia are marked in red with year of detection in parentheses and indicated figure of the host. GenBank accession numbers for isolates used in the analysis are specified where appropriate. WNV genetic lineages suggested by Rizzoli et al. [84] are indicated on the right. Lineages 5 and 7 could not be included in the analysis due to only partial sequence availability. Supporting (≥50%) bootstrap values of 1000 replicates are displayed at the nodes. Horizontal distances are proportional to genetic distance. Scale bar indicates nucleotide substitutions per site.

Although serologic evidence of WNV in Croatia dates back to the 1970s [15], the first seven human clinical cases of WNV neuroinvasive disease were reported in 2012 in eastern Croatian counties bordering Serbia. Acute asymptomatic WNV infections in sentinel horses demonstrated by detection of IgM antibodies preceded human cases. Furthermore, an increased WNV IgG seropositivity in horses (8.7%) compared to 2011 (3.43%) was notified in counties where human cases occurred [6,45]. During the 2013 outbreak, a total of 20 cases were reported in three northwestern counties [7]. From 2014 to 2016, only sporadic human cases were notified, followed by a small outbreak in 2017 (8 cases). However, acute asymptomatic infections and seropositivity in horses were continuously notified [46]. In 2018, Croatia reported the largest WNV outbreak with more than 60 cases of WNV neuroinvasive disease and WNV fever in 11 continental counties. For the first time, fatal WNV infections were detected in a female and a male goshawk (*Accipiter gentilis*) from the same aviary as well as a serologic evidence of WNV infection in one buzzard (*Buteo buteo*) in northwest Croatia [44]. After the first report of human WNV infections, entomological surveys were continuously performed. During the 2012 outbreak, mosquitoes were sampled within the area of WNV neuroinvasive human infections in three northeastern counties. All tested *Culex pipiens* complex pools were negative for WNV RNA [83]. In the period from 2015 to 2020, mosquitoes were collected in Zagreb and its surroundings. So far, none of the tested *Cx. pipiens* and *Aedes albopictus* mosquito pools were positive for WNV [44,47]. WNV seropositivity in poultry was continuously recorded from 2012 in continental Croatian counties. No one human WNV infection was detected in the 2019 and 2020 transmission season. According to genetic lineages suggested by Rizzoli et al. [84], phylogenetic analysis showed that strains detected in humans and goshawk belong to WNV lineage 2 (Figure 3) [44,77,85]. The presented results confirmed active circulation and endemic presence of WNV in continental Croatia. So far, there are no reported WNV cases at the Croatian littoral.

### 2.4. Usutu Virus

**Figure 4:**
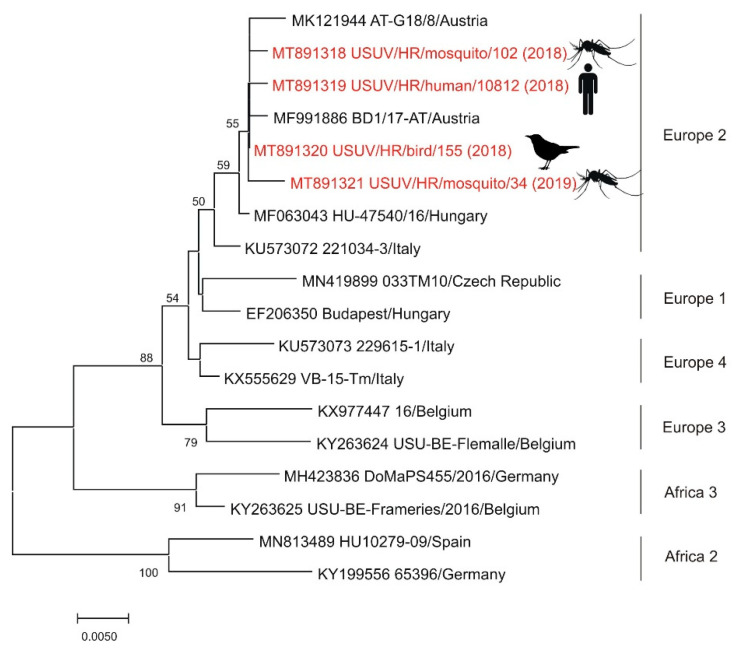
Phylogenetic neighbor-joining analysis of a 543-nucleotide fragment of the USUV NS5 gene. Strains detected in Croatia are marked in red with year of detection in parentheses and indicated figure of the host. GenBank accession number for each isolate used in the analysis is specified. USUV genetic lineages suggested by Cadar et al. [86] are indicated on the right. The highly divergent Africa 1 genetic lineage was not included in the analysis in order to increase the resolution of the phylogram. Supporting (≥50%) bootstrap values of 1000 replicates are displayed at the nodes. Horizontal distances are proportional to genetic distance. Scale bar indicates nucleotide substitutions per site.

The exposure to USUV was documented for the first time in 2011. Two seropositive horses were found in the Sava River Basin, northwestern Croatia [48]. In the following year, the first seropositive human was detected in eastern Croatia [49]. The first three clinical cases of USUV neuroinvasive disease were notified during the 2013 WNV outbreak in Zagreb and its surroundings [7,8], followed by additional three cases detected during the largest WNV outbreak in Croatia in 2018 [44]. In 2018, USUV was detected for the first time in one dead blackbird (*Turdus merula*) from northwestern Croatia. Additionally, USUV-positive mosquito pools were detected in 2016 (*Ae. albopictus*), 2017 (*Cx. pipiens*), 2018 (*Cx. pipiens*), and 2019 (*Cx. pipiens*), respectively in northwestern regions. According to genetic lineages suggested by Cadar et al. [86], sequenced strains from a fatal human case (2018), blackbird (2018) and two *Cx. pipiens* pools (2018, 2019) showed USUV Europe 2 lineage (Figure 4) [44,76,77].

### 2.5. Sandfly Fever Viruses

Sandfly fever is a neglected disease since its reporting is not mandatory in Croatia. Due to mild symptoms in most patients, the number of cases is underestimated and underreported. SFSV and SFNV seropositive persons are detected in both continental parts and the Croatian littoral. Large seroprevalence studies were conducted in 1975–1976 in Croatia which showed very high seroprevalence rates of 62.1% on Brač Island and 59.4% on Hvar Island. The SFNV prevailed; it was far more frequent than double infections and the infections with the SFSV only [15]. Seroepidemiological studies from the 1980s showed an SFNV seroprevalence rate of 23.6% among Adriatic coast residents. Seropositive persons were detected all along the coastline, from Istria in the north to Dubrovnik in the south as well as on islands. Seroprevalence varied according to the region (5.9–45.0%) and increased with age from 4.5% to 51.8% at the age of 40–50 years [54]. A more recently conducted study (2017–2018) found the overall seroprevalence rate of 2.3% and 3.3% for SFSV and SFNV, respectively [55]. Further studies are needed to determine the prevalence of sandfly fever viruses in Croatia.

### 2.6. Toscana Virus

**Figure 5:**
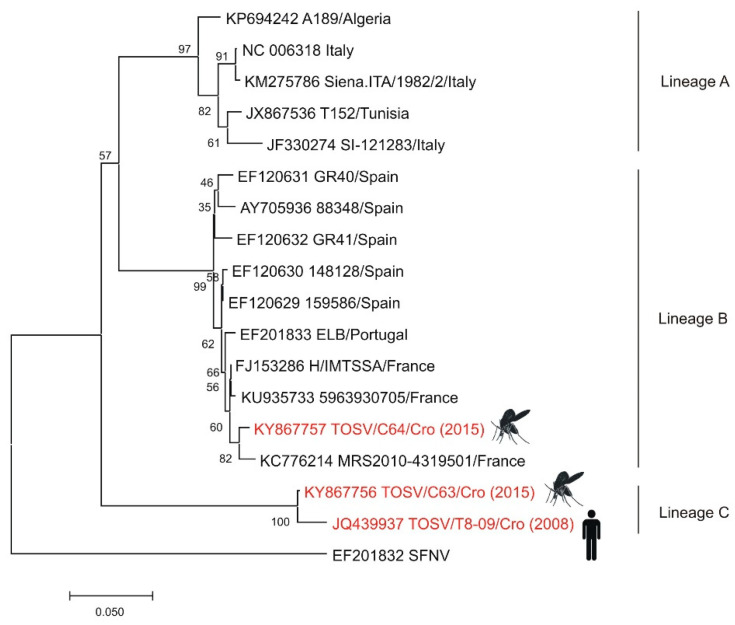
Phylogenetic neighbor-joining analysis of a 321-nucleotide fragment of the TOSV nucleoprotein gene. Strains detected in Croatia are marked in red with year of detection in parentheses and indicated figure of the host. GenBank accession number for each isolate used in the analysis is specified. SFNV was included as an outgroup. Supporting (≥50%) bootstrap values of 1000 replicates are displayed at the nodes. Horizontal distances are proportional to genetic distance. Scale bar indicates nucleotide substitutions per site.

Although high seroprevalence rates for TOSV were detected among residents of the Croatian littoral, TOSV is still a neglected virus in Croatia with only a few documented cases of human neuroinvasive infection. In 2007–2008, cerebrospinal fluid and serum samples were collected from 30 hospitalized patients with aseptic meningitis. TOSV IgM antibodies were detected in four patients, while two had detectable TOSV RNA in the CSF. Phylogenetic analysis of partial L and S segments suggests that TOSV from Croatia represented an autochthonous strain [12]. From 2007 to 2009, a seroprevalence study was conducted. IgG antibodies to TOSV were detected in 37.5% persons: 53.9%, 33.6%, and 6.1% residents of the islands, coastal area, and the mainland of Croatia, respectively. Risk factors significantly associated with TOSV seropositivity were living on islands or in coastal areas and older age. As people from the mainland often travel to the littoral for summer vacations, the possibility of being infected there is not excluded [11]. In the 2018 and 2019 transmission seasons, three additional cases of neuroinvasive infection were also confirmed serologically at the Croatian littoral [13]. In 2015, an entomologic study was conducted. A total of 1453 sand flies were collected from five locations in Croatia. Co-circulation of two TOSV lineages (B and C) was detected in *Phlebotomus neglectus* sandfly pools (Figure 5) [50]. Since TOSV testing is not a part of routine diagnostic algorithms, the true prevalence of the disease is unknown. 

### 2.7. Chikungunya and Zika Viruses

Only one study conducted in 2011–2012 among randomly selected inhabitants of seven Croatian counties located on the Adriatic Coast assessed the seroprevalence to CHIKV. IgG antibodies were found in 0.9% (9/1008) of the tested participants with seropositivity from 0.5% to 1.8% according to region. All mosquito pools tested negative for CHIKV RNA using a real-time RT-PCR [87]. First imported clinically manifested chikungunya fever in Croatia was reported in 2016 in a traveler returning from Costa Rica [88]. After that, sporadic imported clinical cases as well as CHIKV IgG seropositive persons were continuously documented. 

The first imported ZIKV infection case in a Croatian traveler returning from Brazil was described in 2016 [89]. Thereafter, a total of 224 returning travelers were tested for the presence of ZIKV. Acute infection was confirmed in six patients. Although Croatia is not an endemic area for ZIKV, due to the establishment of *Ae. albopictus* in the whole country, importation of virus by returning viremic travelers or tourists could result in a local disease transmission. Therefore, permanent vector control measures should be regularly performed.

### 2.8. Tahyna and Čalovo Viruses

There is only one seroprevalence study on TAHV in Croatia conducted in the 1970s. TAHV antibodies were detected in 7.9% of persons from the northeast Croatia and 0.4% of persons from Dalmatia and the southern Adriatic [14]. As a part of the project CRONEUROARBO, CSF and urine samples from 284 patients with the neuroinvasive disease were tested in 2017–2018; however, no one sample tested positive for TAHV RNA. 

Čalovo virus was isolated in 1969 from a pool of *Anopheles maculipennis* s.l. mosquitoes in Marino Selo village in eastern Croatia [58]. To obtain the information of the level of human and animal exposure to the virus, inhabitants and horses from the same region were tested by the virus neutralization test. While 27.8% of horse serum samples tested positive, all human samples were negative [15,59]. Serum samples from some domestic animals other than horses in the regions along the banks of the Neretva River were also tested. The highest percentage of seropositive animals was detected among cows (35.71%), followed by goats (27.27%) and donkeys (12.50%), while no horse or sheep tested positive [15]. Additionally, hemagglutination inhibiting antibodies was detected in 0.6% of residents of northeast Croatia (around the place of isolation) and 0.5% in Dalmatia and the south part of the Adriatic Sea. Unlike the TAHV seroprevalence, those for the Čalovo virus seem to be similar in the northern and southern Croatian regions [60]. There are no recent investigations on the Čalovo virus in Croatia.

### 2.9. Bhanja Virus

BHAV was isolated in 1974 from *Hemaphysalis punctata* (Canestrini and Fanzago, 1878) ticks collected on the Brač Island. The first human laboratory infection with BHAV was reported in the same year. The role of BHAV as a zoonotic virus was established in 1975, after detection of BHAV by retrospective testing of serum samples of a patient presented with meningoencephalitis and spastic quadriparesis in Zagreb. Three weeks before the symptom onset, the patient had stayed in the northern part of Croatia where the *H. punctata* ticks were found [56]. Subsequently, two laboratory infections occurred in 1977 [16]. Seroepidemiological studies conducted in 1970 on Croatian islands showed neutralizing antibodies in 31.5% (11.6–61.3%) inhabitants of the Brač Island, 2.2% of inhabitants of the islands around Zadar, and 1% of inhabitants of the Hvar Island. No seropositive person was detected on the Mljet Island. In addition to Dalmatia, 7.1% of seropositive persons were also detected among inhabitants of northern Croatia [56]. In the 1980s, BHAV hemagglutination inhibiting antibodies were detected in 3.3% of dogs from regions near the Hungarian border [57]. As a part of the project CRONEUROARBO, CSF and urine samples from 284 patients with the neuroinvasive disease were tested; however, no sample tested positive for BHAV RNA. Further studies are needed to determine the (sero)prevalence of this neglected arbovirus in Croatia.

## 3. Mosquito Surveillance in Croatia

Among 52 mosquito species which were recorded so far on Croatia’s territory, the special attention in recent years has focused on invasive mosquito species, especially the Asian tiger mosquito *Ae. albopictus* (Skuse, 1894), which meets the criteria for transmission of the DENV, CHIKV, and ZIKV [90]. The first detection of *Ae. albopictus* was in Zagreb in 2004 [91]. Over the next years, *Ae. albopictus* has been detected and then established in numerous places in coastal Croatia and islands [92,93]. The spread and establishment of the species have also been recorded in northwestern continental areas [90], as well as in the eastern part of the country [94]. Another invasive species *Aedes japonicus japonicus* (Theobald, 1901), was first recorded in 2013 in Ðurmanec and Macelj (Krapina-Zagorje County, northwestern Croatia). In 2014 and 2015, several counties further to the east were included in the survey, leading to the detection of *Ae. j. japonicus* approximately 100 km eastward from the initially surveyed region [95,96]. Following the Croatian Institute of Public Health (CIPH) initiative, monitoring of invasive mosquito species in Croatia started at the national level in 2016 and has been carried out continuously since then. The aim was to collect data on species distribution, creating a distribution map and a unique national database to conduct a risk assessment for mosquito-borne diseases [97]. The framework for mosquito surveillance in Croatia is set by the legislation at the local and national level [98]. CIPH in cooperation with the County Public Health Institutes (CPHI) created the protocol for monitoring of invasive mosquito species. The monitoring has been conducted using ovitraps method at possible entry points of invasive mosquito species (cemeteries, petrol stations, tyre storage areas, tyre repair shops, truck parking areas, border crossing) and house yards. The monitoring has been carried out by CPHI (*n* = 21) and Department of Biology, University of Osijek. Conducted national monitoring confirmed the presence of *Ae. albopictus* in all Croatian counties. In addition, monitoring has confirmed the expansion of *Ae. j. japonicus* in most continental counties [99]. In the areas where invasive mosquito species are established, priority is set on integrated methods of mosquito control and surveillance of disease, as well as prevention of further vector spreading. Mosquito control at the national level provides the necessary information for planning timely responses to disease outbreaks, developing entomological capacities, strengthening multidisciplinary collaboration, and approaching the implementation of better prevention and response to mosquito-borne diseases [100]. Additionally, in several counties (Zagreb, Osijek-Baranja County, Istria County and Split-Dalmatia County), a monitoring of native mosquito species (*Anopheles, Aedes, Ochlerotatus, Culex*) has been conducted. 

## 4. Hard Ticks as Vectors of Zoonotic Pathogens in Croatia 

Hard ticks (Acari: Ixodidae) are medically the second most important group of haematophagic arthropods after mosquitoes [101]. In Croatia, in the second half of 20th century, the comprehensive studies on the tick fauna were carried out along the Adriatic coast and islands in the Adriatic Sea, as well as in northwestern parts of the country. Despite many faunistic studies, some areas have not yet been sufficiently studied; one area was eastern part of Croatia. Within the project CRONEUROARBO, a zoologic study was performed (2018–2019) in 49 localities in eastern Croatia. A total of 2889 ticks were collected and classified into seven species and three genera. *Ixodes ricinus* (Linnaeus, 1758) was the most abundant tick species recorded in 45 localities [102,103]. These data of high abundance of species *Ix. ricinus* deserve attention since this tick is the main vector of TBE virus (TBEV) [42]. In addition, in two localities, *Ixodes hexagonus* (Leach, 1815) was recorded [102]. This species has recently been recognized as the second vector of TBE virus in Croatia indicating high vector potential of this species in continental regions [42]. A total of 22 species of hard ticks classified in five genera have been recorded in Croatia. *Ixodes* Latreille, 1795 is the best-represented genus with eight species recorded. Genus *Haemaphysalis* Koch, 1844 is represented by six species, followed by genus *Rhipicephalus* Koch, 1844 with four species. Genera *Dermacentor* Koch, 1844 and *Hyalomma* Koch, 1844 are represented by two species each [104]. Due to the high diversity of tick fauna in Croatia and the ability of ticks to transmit numerous pathogens, many tick species need to be continuously monitored at a local and national level because the spreading of ticks and their pathogens are connected by series of human activities such as globalization of the economy, increased human movement, international animal movements, habitat changes, and deforestation.

## 5. Rodent-Borne Viruses

### 5.1. Lymphocytic Choriomeningitis Virus

Lymphocytic choriomeningitis remains a neglected disease in Croatia, with only a few published studies on the seroprevalence of LCMV in humans. Two studies were conducted in 2006 in limited geographic areas and specific population groups. A study conducted among forestry workers in Posavina (region of the Sava river basin) showed a seropositivity of 5.1% [61]. The other study conducted in the rural population of Vir, a small island at the Croatian littoral which is an endemic region for murine typhus showed a very high seroprevalence rate of 36%, one of the highest LCMV human prevalence rates reported worldwide. The high seroprevalence is probably due to the combination of several factors: the isolated nature and small size of the island (22 km^2^), the autochthonous inhabitants, the existence of rural communities as well as the close contact with rodents [62]. In a more recent study (2016–2017), LCMV seroprevalence was analyzed in inhabitants of continental Croatian regions. Serum samples of professionally exposed (forestry workers, hunters, agriculture workers in contact with rodents) and non-exposed population groups (general population, pregnant women) were tested. LCMV IgG antibodies were detected in 6.8% of participants, 9.8% exposed persons, and 5.1% non-exposed persons. No participant was LCMV IgM positive. Although higher seropositivity was found in males (8.9%) compared to females (4.7%), inhabitants of suburban/rural areas (9.2%) compared to inhabitants of urban areas (4.6%) and persons who used well as a source of water (11.4%) compared to those who used tap (5.6%), these differences were not significant. Contact with rodents in the house/yard and cleaning rodent nests correlated strongly with the LCMV seroprevalence [17]. As a part of the project CRONEUROARBO, CSF samples of patients with the neuroinvasive disease were tested for the presence of LCMV RNA; however, no one sample tested positive. Human clinical cases of neuroinvasive LCMV infection have not been reported in Croatia.

### 5.2. Hantaviruses

**Figure 6:**
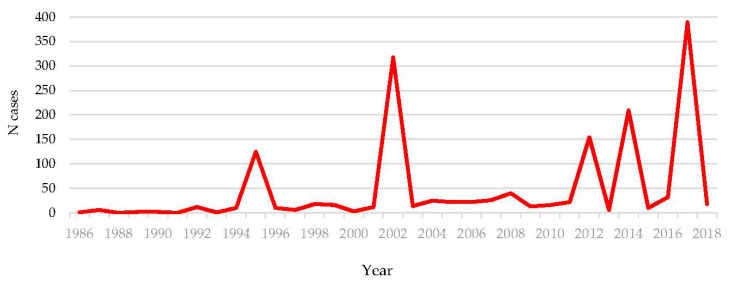
Number of reported HFRS cases in Croatia (1986–2018).

Sporadic cases as well as hantavirus outbreaks are reported regularly in Croatia. Since 2002, all of Croatia except for the coastal region and the islands is endemic for hantaviruses [18,19,105,106]. Several large outbreaks were notified in recent years: 2012 (154 cases), 2014 (2009 cases), and 2017 (389 cases) (Figure 6) [18,21,22,107]. Data on risk activities collected during the largest outbreak in 2017 involving 15/21 Croatian counties showed that 99.6% of cases from Zagreb reported hiking in recreational areas as the main risk activity. In other Croatian counties, 48% of cases reported farm/forestry work, and 26% cleaning house or surroundings [108]. Several studies analyzed the prevalence of hantaviruses in rodents. In 1995, small mammals were collected from Camp Pleso, as part of a survey initiated to determine the extent of rodent infestation and evaluate military personnel’s health risk. In 13.2%, *Microtus arvalis* and *Microtus agrestis*, TULV was detected [109]. In 2003–2004, rodents were collected during two expeditions, in the northeastern (2003) and the southwestern Croatia (2004). Six of the 6.3% of hantavirus antigen positive *Apodemus* mice were genotyped as DOBV. In addition, one SAAV strain was detected [23]. In 2007, hantavirus RNA was confirmed in 52% of rodents: DOBV in 71% of *A. flavicollis* and PUUV in 19% of *M. glareolus*. Hantavirus antibodies were detected in 43% of rodents: anti-SAAV/DOBV in 57% of *A. flavicollis* and anti-PUUV in 19% of *M. glareolus* [24]. A study conducted in nonepidemic periods in two different sites in Croatia (mountainous and lowland region) showed antibodies against hantaviruses in 25.5% rodents from the mountainous area. Additionally, 21.3% of samples from the mountainous area and 29.0% from the lowland area yielded positive results for either PUUV or DOBV using RT-PCR. Phylogenetic analyses revealed two distinct genetic subclusters of the Croatian PUUV and DOBV strains [63]. High infection rate (77.4%) of *Myodes glareolus* with PUUV was associated with a winter HFRS outbreak on Medvednica mountain in 2012, while DOBV was detected in 9.8% *A. flavicollis* [107]. Hantaviruses PUUV and DOBV are widely distributed viruses, with human cases occurring every year in continental Croatian regions. Although SAAV was detected in rodents, not a single SAAV sequence has been recovered from patients with hemorrhagic fever with renal syndrome so far in Croatia.

## 6. Rodent Control and Monitoring in Croatia

**Figure 7:**
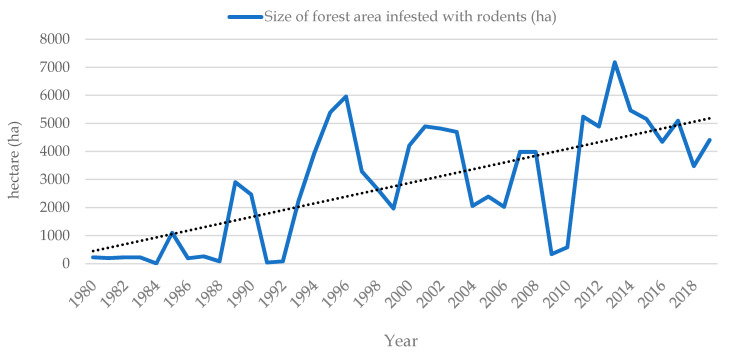
Monitoring of small rodents in Croatian state-owned forests (1980–2018).

Rodents are the most abundant and diversified order of living mammals that inhabit almost every type of land ecosystem worldwide [110,111,112]. The most common rodent species that can cause damage in natural and in urban ecosystems in Croatia are striped field mouse (*Apodemus agrarius* Pallas, 1771), yellow-necked field mouse (*Apodemus flavicollis* Melchior, 1834), wood mouse (*Apodemus sylvaticus* Linnaeus, 1758), bank vole (*Myodes glareolus*, Schreber 1780), common vole (*Microtus arvalis* Pallas, 1779), field vole (*Microtus agrestis* Linnaeus, 1761), European water vole (*Arvicola amphibius*/*terrestris*/Linnaeus, 1758) and European pine vole (*Microtus subterraneus* de Sélys-Longchamps, 1836.), house mouse (*Mus musculus* Linnaeus, 1758), brown rat (*Rattus norvegicus* Berkenhout, 1769), black rat (*Rattus rattus* Linnaeus, 1758) [113,114]. Damage caused by rodents, among many other factors, depends on their abundance. Therefore, getting familiar with their seasonal and multi-annual dynamics is a prerequisite for the efficient implementation of any rodent management system [115,116]. Monitoring of small rodents in state-owned forests started some 40 years ago as one of the annual activities of the Diagnostic and Prognostic Service of the Croatian Forest Research Institute (Figure 7) [117]. 

From 1980 to 2016, monitoring consisted of recording infested forest areas (ha) with noticeable rodent damage (on stem and roots of forest tree saplings). In 2017, monitoring got improved by Integrated Pest Management (IPM) principles [118,119], so now it involves sampling rodents by using snap traps on line-transects [120] and also identifying relative share of damaged forest seeds and seedlings primary at young regeneration forest stands. Results of such monitoring are being entered and processed through online database (https://stetnici.sumins.hr/). Rodent monitoring does not include testing sampled individuals for pathogens.

From the beginning of the 1980s, forest areas with noticeable rodent activity and damage continue to increase. Since then, rodent populations typically reached their peaks every 3–4 years, but, during the last ten years, outbreaks have tended to occur more frequently, in 2–3-year cycles, which corresponds to trends in different parts of western and central Europe (Germany, Belgium, etc.) explained by the ongoing global climate change [121,122]. Traditional approaches to rodent management in Croatia rely on the direct reduction of the rodent population using rodenticide baits (1st and 2nd generation anticoagulants). According to the List of registered plant protection products [123], the only rodenticide that can be used in forestry and agriculture in Croatia for plant protection is fumigant zinc phosphide. In public health and public hygiene, conditions for the implementation of disinfection, fumigation and pest control measures are regulated by legal provisions in force in the Republic of Croatia issued by the Ministry of Health, Ministry of Agriculture and Veterinary Directorate. A list of currently approved active substances allowed in biocides that can be used in rodent control includes 1st, 2nd, and 3rd generation anticoagulants with active substance: coumatetralyl, brodifacoum, bromadiolone, difenacoum, flocoumafen, difethialone, chlorophacinone, and warfarin [114,124]. Undoubtedly, rodents, such as pests and zoonotic reservoirs, continue to contribute to the human diseases and production losses. Due to climate change and conversion of natural habitats to agricultural or urban ecosystems, rodents will most likely be harboring undiscovered zoonotic pathogens in the future [125,126]. Improvement in rodent management systems, both in public health and plant protection, that we need to implement is ecologically-based rodent management (EBRM) approach that would rely less on rodenticides and more on community-wide habitat management principles [127,128]. 

## 7. Respiratory Viruses

### SARS-CoV-2

The first case of coronavirus disease (COVID-19) was detected in Croatia on February 25, 2020. A total of 195,728 cases and 3257 deaths from COVID-19 were reported by December 22, 2020 (data of the CIPH). Several seroepidemiological studies on COVID-19 have been conducted in Croatia. In April 2020, SARS-CoV-2 antibodies were detected in 1.27% of industry workers in two counties at the Croatian littoral [33]. A similar seroprevalence rate of 1.5% was found among personnel of the healthcare facilities from counties with a high incidence of COVID-19 tested from April 25 to May 24, when the COVID-19 epidemic curve was approaching the end of the first wave in Croatia [32]. Additionally, SARS-CoV-2 IgG antibodies were detected in 2.9% of children and 2.5% adults from the Zagreb area [129]. Preliminary results showed higher seroprevalence rates in hemodialysis and transplant patients (5.8% and 19.4–20.7%, respectively) [130]. From 26 February 2020, just one day after the first confirmed human COVID-19 case, to 15 June 2020, 656 dog and 131 cat serum samples were collected from animals admitted to three veterinary facilities in Croatia. Neutralizing antibodies were confirmed in 0.76% of cats and 0.31% of dogs. Furthermore, on 25 May 2020, a total of 122 serum samples from employees of the Faculty of Veterinary Medicine University of Zagreb were collected. ELISA reactivity was recorded in 5.19% of administrative, basic and preclinical sciences department personnel and 5.13% of animal health service providers and laboratory personnel; however, neutralizing antibodies were not confirmed in any of the human samples. A small number of seropositive animals suggest that infections are rare and are following infections in the human population [34]. So far, there have been no published data on the genetic characterization of SARS-CoV-2 strains detected in Croatian COVID-19 patients. The research on the circulation of SARS-CoV-2 in wildlife species and the environment has recently started. The preliminary data suggest the circulation of SARS-CoV-2 in bats, wild boars, and sewage samples. Bivalves, surface waters, and gulls were negative. The focus of the ongoing research is the implementation of serological and molecular confirmatory methods [131].

## 8. Food-Borne and Enteric Viruses

### 8.1. Hepatitis E Virus

The autochthonous HEV infection in Croatia was documented in 2012 [132]. Since then, several epidemiological studies have been conducted in different population groups (Table 2). In patients with liver-related pathology, HEV has been tested in hepatitis patients negative for acute viral hepatitis A-C showing 10.7% of anti-HEV IgM or IgG antibodies [64], and in adult patients with different chronic liver diseases detecting previous exposure (IgG) in 15.1% of patients, with 4.5% of them being IgM positive, but not a single HEV RNA positive [133]. A pilot study conducted in different population groups (2016) showed lower overall HEV IgG seropositivity of 5.6%, with 1.9% being IgM positive and without HEV RNA positivity. The IgG prevalence ranged from 2.7% in healthcare professionals, 6.1% in injecting drug users, 8.6% in war veterans to 8.9% in alcohol abusers. The HEV IgG positivity increased with age and increased household members, whereas the area of suburban/rural residence has been identified as the main risk factor for HEV seropositivity [25]. Low previous exposure has also been documented in pregnant women (2.9%) [134]. However, these results contrast with a study among Croatian voluntary blood donors, which showed a high overall IgG seropositivity rate of 21.5% with varying rates among the counties (7.5–50.3%). The age-related effect has been evident, with a higher HEV seropositivity in blood donors ≥40 years [135]. Professionally exposed persons, such as hunters and forest workers, have HEV IgG seropositivity ranging from 4.0 to 8.1% [136]. The patients with end-stage kidney disease on hemodialysis have the highest overall IgG seroprevalence rate (27.9%) in the country, with significant variations between dialysis centers ranging from 5.2% (coastal in the South) to 43.4% (continental in the North). Older age (>60 years), living in the continental regions, and transfusion of blood products have been associated with IgG seropositivity [27]. In the immunocompromised population, HEV has been tested in HIV-infected patients showing HEV IgG seropositivity of 1.1% [64] and adult liver recipients with HEV IgG seroprevalence rate of 24.4%. Female gender, older age, and sewage system connected to a septic tank have been identified as risk factors for HEV seropositivity, whereas the highest level of education has shown to be a protective factor [26]. HEV infection has been reported in Croatian pigs and wild boars from 2009 onwards and they have proven to be reservoirs of HEV [65,66]. Recently, HEV genotype 3a has also been derived from a naturally infected yellow-necked mouse [67]. Phylogenetic analysis showed that HEV strains derived from humans, swine, wild boars, and the yellow necked mouse in Croatia belong to the *Orthohepevirus* A group, genotype 3 and clustered within four subtypes (3a, 3c, 3e and 3f), with the majority belonging to subtypes 3a and 3c that are considered endemic in Croatia. All strains regardless of origin show a high genetic relationship within each subtype [68].

### 8.2. Rotaviruses

**Figure 8:**
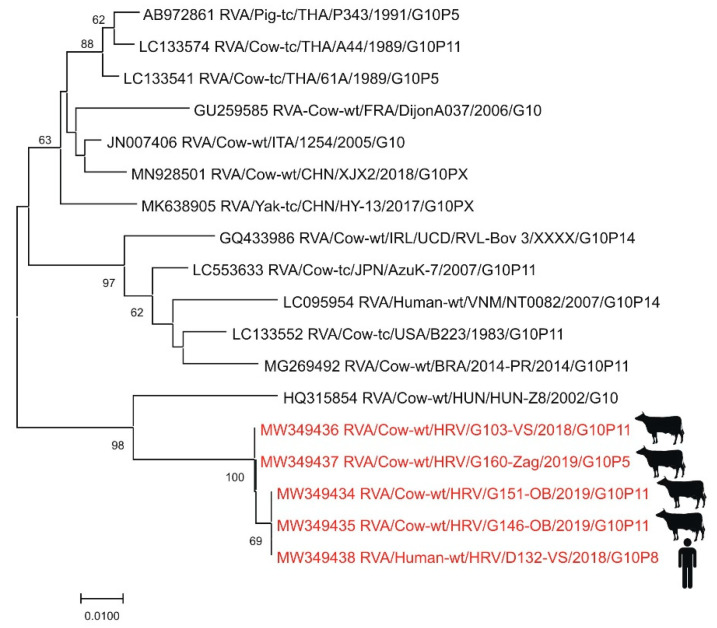
Phylogenetic neighbor-joining analysis of a 286-nucleotide fragment of the VP7 gene of G10 RVA strains. Strains detected in Croatia are marked in red with the indicated figure of the host. GenBank accession number for each isolate used in the analysis is specified. Supporting (≥50%) bootstrap values of 1000 replicates are displayed at the nodes. Horizontal distances are proportional to genetic distance. Scale bar indicates nucleotide substitutions per site.

Only a few studies reported RVA prevalence and genotypes in humans in Croatia since 2008 when a notification of rotavirus infection became mandatory [137]. The investigation on animal RVAs and RVAs present in the environmental samples has started only recently [69]. First data on the prevalence of certain RVA genotypes in humans (mostly children under five years of age from Zagreb) were reported for the seasons 2005 and 2006 (*n* = 459) with a dominance of typical human genotypes (G1–G4, G9). In both years, two possible human-animal reassortant strains (G8 and G10) were detected. Of particular interest was the large share (19.7%) of the genotype G8 which emerged in 2006 [138]. The whole-genome sequencing of the RVA strain responsible for the 2006 outbreak revealed the Wa-like genotype constellation G8-P [8]-I1-R1-C1-M1-A1-N1-T1-E1-H1. This strain was closely related in the VP7 segment to human G8 strains from Africa. Even though G8 genotype is considered a typical bovine genotype, the human strain from the 2006 outbreak was distantly related to bovine strains [139]. Another molecular epidemiology study was conducted on RT-PCR positive samples (*n* = 822) originating from three hospital centers in Čakovec, Zagreb and Split (children under five years of age) between 2012 and 2014. The prevalence of common human genotype combinations was 88.8% with the most common genotype combination G1P [8] (61.9%) [28], similar to other European countries at that time [31]. The interesting finding is the high prevalence of human–human reassortants (7.7%) and detection of genotype G6 in two samples, which is considered a typical bovine genotype [28]. The preliminary results of the most recent study on human RVAs, which started in 2018 [29], indicate a dominance of genotype G3 (55.1%) and the emergence of equine-like G3P [8] and G1P [8] intergenogroup reassortant strains [140]. The whole-genome sequence of selected equine-like G3P [8] strains has confirmed a DS1-like genotype constellation G3-P [8]-I2-R2-C2-M2-A2-N2-T2-E2-H2, which is not typical for common human G3 strains [141]. The rising prevalence of G3P [8] strains has been documented in recent years throughout Europe. Simultaneously, a continuous decline in G1P [8] strain incidence was observed, suggesting a possible cross-border impact of increased vaccinated cohorts against RVA across Europe [142]. Further evidence of the circulation of human–animal reassortant strains in Croatia has been provided by the sporadic detection of genotypes G6, G8, G10, and P [14], typical for bovines [29]. The zoonotic background of autochthonous RVA strains was especially evident for genotype G10 (Figure 8) [140]. The concurrent study on animal and environmental RVAs has discovered the high genetic heterogeneity of circulating strains in different domestic and wild species of animals with frequent detection of RVAs in bivalves and sewage/surface waters [29].

## 9. Other Zoonotic Viruses

### Rabies

The last case of human rabies in Croatia was detected in 1964 [35]. Silvatic rabies has been endemic since 1977, with the red fox (*Vulpes vulpes*) as the main disease reservoir and vector [70]. Although the RABV is mostly maintained within the fox population, foxes occasionally transmit the virus to other wild and domestic animal species [71]. Between 1977 and 2010, RABV was detected in 15.5% of tested domestic and wild animals. Red foxes were the most commonly infected species (23.6%), followed by dogs (2.7%) and cats (2.4%). The number of positive cases decreased sharply since 2011 as a result of ORV of foxes. ORV campaigns have been conducted twice a year since the spring of 2011, and from the autumn of 2012, the program was extended to the entire country [36]. The ORV results were outstanding: while there were 11.1% rabies positive samples in domestic animals and wildlife in 2010, this number by 2015 was reduced to 0% [72]. Between 2016 and 2017, a study on the prevalence of lyssaviruses in Croatian bat populations from four continental and seven Mediterranean locations was performed. A total of 455 bats belonging to seven species (*E. serotinus, Myotis blythii, Myotis emarginatus, Myotis myotis, Myotis nattereri, Miniopterus schreibersii*, and *R. ferrumequinum*) were captured through active surveillance. Lyssavirus antibodies were documented in 5.71% bats. The majority of seropositive bats were found in one cave in eastern Croatia, and most seropositive bats belonged to *Myotis myotis*. All oropharyngeal swabs tested negative for the lyssavirus. This study confirmed the presence of EBLV-1 antibodies in bat populations for the first time in Croatia [36,73].

## 10. Conclusions

In the past decade, the incidence of (re-)emerging viral infectious diseases has increased and represents a significant threat to global health. The majority of emerging pathogens are of zoonotic origin. In addition, many other zoonotic viruses remain neglected. The epidemiology of zoonotic viruses is continuously changing due to the virus spreading into new areas, vectors, and hosts. Therefore, the knowledge regarding the epidemiology, diagnostics and treatment of these pathogens needs continuous updating.

Like in many other European countries, (re-)emerging flaviviruses (TBEV, WNV, USUV) are widely distributed in both humans and animals in Croatia. TOSV is still a neglected arbovirus with an underestimated number of cases. While some rodent-borne viruses (PUUV, DOBV) are endemic in continental Croatian regions, LCMV is a neglected virus confirmed only serologically. HEV is an emerging virus of zoonotic importance in Croatia with high seroprevalence in some population groups. A novel coronavirus, SARS-CoV-2, has been confirmed in humans and pet animals, while preliminary data suggest the presence of the virus in bats, wild boars, and sewage samples as well.

Introduction and implementation of surveillance in humans, animal reservoirs, and vectors (‘One Health’) point out the importance and efficiency of multidisciplinary collaboration in the diagnosis and control of zoonotic viruses in Croatia. The strengths of these programs are early detection of virus circulation, timely reporting to human health service institutions and local authorities in order to inform the local communities and for establishing the vector control and preventive measures for human health protection [46].

## Figures and Tables

**Table 1:** The most important zoonotic viruses detected in Croatia.

Virus	Humans	Animals	Arthropod Vectors	References
Arboviruses				
TBEV	Clinical cases, serology, virus detection *	Horses (serology), dogs (serology), goats (serology), deer (virus detection *)	Virus detection *	[2,4,38,39,40,41,42,43]
WNV	Clinical cases, serology, virus detection *	Horses (acute asymptomatic infections, serology), birds (clinical cases, serology, virus detection *), poultry (serology)		[6,7,44,45,46]
USUV	Clinical cases, serology, virus detection *	Horses (serology), birds (virus detection *)	Virus detection *	[7,8,44,47,48,49]
TOSV	Clinical cases, serology, virus detection *		Virus detection *	[11,12,13,50]
DENV	Clinical cases, serology, virus detection *			[5,51,52,53]
SFSV/SFNV	Serology			[15,54,55]
TAHV	Serology			[14]
BHAV	Clinical cases **, Serology	Dogs (serology)	Virus isolation	[16,56,57]
Čalovo	Serology	Horses, cows, goats, donkeys (serology)		[15,58,59,60]
Rodent-borne viruses				
LCMV	Serology			[17,61,62]
PUUV	Clinical cases, serology	Rodents (virus detection *, serology)		[18,19,21,22,23,24]
DOBV	Clinical cases, serology	Rodents (virus detection *, serology)		[19,21,22,63]
SAAV		Rodents (virus detection, serology)		[23,24]
TULV		Rodents (virus detection)		[23,24]
Other zoonotic viruses				
HEV	Clinical cases, serology, virus detection *	Swine (virus detection *, serology), wild boar (virus detection, serology), yellow-neck mouse (virus detection)		[25,26,27,64,65,66,67,68]
Rotaviruses	Clinical cases, virus detection *	Cattle and swine (clinical cases, virus detection), dogs, red foxes, European jackals, red deer, roe deer, wild boar and gulls (virus detection)		[28,29,69]
SARS-CoV-2	Clinical cases, serology, virus detection *	Dogs (serology), cats (clinical cases, serology)		[32,33,34]
RABV	Clinical cases	Foxes, dogs, cats, bats, horses (virus detection *)		[35,36,70,71,72,73]

* sequencing; ** including laboratory infections; TBEV = tick-borne encephalitis virus; WNV = West Nile virus; USUV = Usutu virus; TOSV = Toscana virus; DENV = dengue virus; SFSV = Sandfly fever Sicilian virus; SFNV = Sandfly fever Naples virus; TAHV = Tahyna virus; BHAV = Bhanja virus; LCMV = Lymphocytic choriomeningitis virus; PUUV = Puumala virus; DOBV = Dobrava virus; SAAV = Saaremaa virus; TULV = Tula virus; HEV = Hepatitis E virus; RABV = rabies virus.

**Table 2:** Hepatitis E (sero)prevalence in different population groups in Croatia.

Population	N Tested	HEV IgM/IgG/RNA Prevalence	References
Liver-related pathologies			
Hepatitis patients negative for acute viral hepatitis A-C	504	IgM/IgG 10.7% HEV RNA 5/14 anti-IgM positive	[64]
Different CLD patients	438	IgG 15.1%; IgM 4.5%; HEV RNA 0%	[64]
Non-liver-related entities			
Healthcare professionals	37	IgG 2.7%	[25]
Alcohol abusers	56	IgG 8.9%	[25]
War-related PTSD patients	35	IgG 8.6%	[25]
Injecting drug users	49	IgG 6.1%	[25]
Pregnant women	68	IgG 2.9%	[134]
Voluntary blood donors	1036	IgG 21.5%; IgM 4.4%; HEV RNA 0%	[135]
Professionally exposed persons			
Forest workers	62	IgG 6.5%	[136]
Hunters	25	IgG 4.0%	[136]
Immunocompromised patients			
HIV-infected patients	88	IgM/IgG 1.1%	[64]
Liver transplant recipients	242	IgG 24.4%; IgM 0.8%; HEV RNA 0%	[26]
HD patients	394	IgG 27.9%; IgM 0.04%; HEV RNA 0%	[27]
